# Rural Caregivers Bridge the Gap between Older Patients and In-Home Occupational Therapy Video Telehealth: A Qualitative Study

**DOI:** 10.20900/agmr20260013

**Published:** 2026-05-28

**Authors:** Megan E. Gately, Dylan E. Waller, Matthew C. Maynard, Lauren R. Moo

**Affiliations:** 1 New England Geriatric Research Education and Clinical Center (GRECC), VA Bedford Healthcare System, Bedford, MA 01730, USA; 2 VA Portland Healthcare System, Portland, OR 97239, USA; 3 Charles Geoge VA Medical Center, Asheville, NC 28805, USA

**Keywords:** occupational therapy, telemedicine, caregivers, veterans, rural health services

## Abstract

**Background:**

In-home video telehealth expanded access to services for older rural Veteran patients, yet many require caregiver assistance. Understanding caregivers’ experiences is essential to optimizing these encounters.

**Objective:**

To explore caregiver perspectives on facilitating in-home occupational therapy video telehealth for older rural Veterans within the US Veterans Health Administration (VHA).

**Methods:**

Semi-structured interviews were conducted with rural caregivers (N = 19) from four VHA sites who had assisted an older Veteran patient with in-home video. Interviews focused on caregivers’ participation before and during sessions, and perceived benefits and drawbacks of video. We employed directed content analysis utilizing a rapid analytic approach, given the time-pressured nature of our project.

**Results:**

Caregivers were predominantly White and female, with an average age of 60; most lived with patients. Five themes emerged: (1) Caregiver Level of Involvement in In-Home Video Telehealth Visits; (2) Experience of Technological Aspects of In-Home Video; (3) Experience of Clinical Aspects of In-Home Video; (4) Beliefs about the Usefulness and Scope of In-Home Video; (5) Recommendations for In-Home Video.

**Conclusions:**

In-home video telehealth expands access to rural older patients, relying heavily on caregivers. It works for routine care but has limits and connectivity barriers, requiring improved procedures and infrastructure.

## INTRODUCTION

Rural Veterans are a growing patient population which faces distinct barriers to care, including older age, multiple complex medical conditions, lower socio-economic status, and limited transportation [1]. Nearly 4.7 million U.S. Veterans live in rural areas, constituting nearly a quarter of the total Veteran population, with 54% aged 65 or older [2]. Rural Veterans from racial and ethnic minority groups and those with disabilities face additional access challenges [3–5]. Older rural Veterans face barriers to geriatrics and specialty care, which tend to be concentrated in urban settings [4,6,7], including occupational therapy, which addresses patients’ day-to-day functional and quality of life needs [8]. To address this gap, the Veterans Health Administration (VHA), the largest integrated healthcare system in the United States, has been offering telehealth for decades through modalities such as store-and-forward, e-consultation, and synchronous video telehealth into patients’ homes and between clinic locations [9–12]. This includes a substantial increase in the use of in-home video telehealth, a live, synchronous encounter in which the Veteran patient receives care in the home by a VHA clinician situated at the medical center, during the COVID pandemic [11,13]. Between 2020 and 2023, in-home video encounters comprised over 90% of VHA telehealth encounters [11], driven in part by VA’s tablet loaner program for Veterans lacking the resources for in-home video telehealth [13–15]. Despite these gains, uptake was higher for younger and urban Veterans [11,16,17], with gaps remaining in use of in-home video telehealth with older and rural Veterans.

Various health and sociodemographic factors limit older rural Veteran ability to access in-home video telehealth services. Older Veteran patients suffer the long-standing effects of service-related injuries ranging from physical disabilities to mental health conditions which can make accessing video more challenging due to factors such as difficulty accessing a private location [6,18–20]. Hearing and vision loss and other sensory impairments may limit use of in-home video, as telehealth devices and platforms are often not adapted for such needs [21–25]. Older Veterans also have higher rates of dementia and other cognitive concerns resulting from toxic exposures and traumatic brain injuries [26,27] which can serve as a barrier to patients’ ability to complete the technological requirements of in-home video telehealth [28]. While caregivers are often cited as important supports to enable patient engagement in in-home video telehealth [29,30], little is known about the caregiver support role for specialty care services like occupational therapy.

Caregivers have a key support role to play in occupational therapy in-home video telehealth services, according to our prior work and the work of others [31,32]. As mentioned above, occupational therapy supports individuals and populations to engage in meaningful daily activities using interventions such as home safety assessments, adaptive equipment training, and therapeutic exercises [33,34]. As the older adult population grows—especially those with multiple health determinant risk factors who experience higher rates of functional limitations—occupational therapy offers an important approach to improving daily functioning and quality of life through support with Activities of Daily Living (ADLs), Instrumental Activities of Daily Living (IADLs), and leisure participation. Related to use of telehealth for occupational therapy, before the COVID-19 pandemic, multiple studies demonstrated that telehealth was comparably effective to in person occupational therapy services across various assessments, interventions, and populations, including adults with complex conditions, children with ASD or other school-based needs, and caregivers of individuals with disabilities [35–39]. A recent systematic review supports adaptation of several occupational therapy services, including group-based lifestyle coaching to promote health and wellness, cognitive-behavioral therapy for chronic pain, exercise interventions, and caregiver education and skills training, to telehealth. Such interventions support patients with chronic conditions, neurological conditions, and those affected by COVID-19 isolation [40]. Occupational therapy practitioners have also utilized telehealth to address home safety [41–44]. Our work from the perspective of occupational therapists themselves indicates an important role for caregivers during in-home video telehealth occupational therapy visits [31,45]. However, there are gaps in our understanding of caregivers’ perspectives about supporting rural older patients to participate in in-home video telehealth occupational therapy visits. Caregiver perspectives about engaging in in-home video for occupational therapy may have translational significance to other specialty care services.

## MATERIALS AND METHODS

### Study Design

The objective of this qualitative project was to gather caregivers’ perspectives about assisting an older rural Veteran patient with an in-home video occupational therapy visit. We chose to interview caregivers given their potential to assist patients with video visits and general role assisting with transportation to, organization of and decisions around patient care. To meet this objective, semi-structured interviews were conducted with family caregivers of patients who resided in rural areas and who had completed an in-home video occupational therapy visit.

### Interview Participants

Caregivers were those who assisted a rural Veteran patient (age 65 or older) with an in-home video occupational therapy visit. Veteran rurality was defined using an algorithm developed Veterans Health Administration [46]. Caregivers were broadly defined as an informal or unpaid friend or family member of the Veteran who participated in an in-home occupational therapy video telehealth session. Caregivers were identified by occupational therapists from across four geographically diverse VHA locations serving Veterans: Chillicothe, Ohio; Asheville, North Carolina; Gainesville, Florida; and, Minneapolis, Minnesota. Occupational therapists at each site were contacted directly via email by the first author who requested that they provide names of patients who had completed a video visit within the last 6 months. If needed, the first author sent a follow-up email. Study team members utilized patients’ electronic health records to confirm eligibility (e.g., patient age and completion of video visit) and to identify contact details.

Potentially eligible participants were then mailed an invitational letter and study FAQ sheet, informing them about the study. Letters were followed by recruitment calls by study team members who first discussed the project with patients and received permission to contact the caregiver, followed by contact with caregivers to invite them to participate. Of 146 referrals received from OTs, 73 were ineligible prior to contact (with the most common reason being Veteran was not rural or was younger than 65), 42 were unreachable or unresponsive, 11 declined to participate (with the most common reason being that they were too busy), and 19 caregivers agreed to participate. Once caregivers agreed to an interview, interviews were either conducted at that time or scheduled for a later date.

### Data Collection

Interviews were conducted by study team members (MG, DW) between March and September 2023. Interviews were designed to last 45–60 minutes and conducted via Microsoft Teams telephone function. The interview guide (see Appendix A) was collectively drafted by the study team (MG, MM, LM, DW). Two pilot interviews were conducted internally within the team to test and fine tune the guide, with no substantial changes needed as a result.

Interviews began with collection of caregiver demographics, including their relationship to the patient followed by questions focused on caregivers’ experience in participating with an in-home occupational therapy video visit. Caregivers were asked to describe their experience and prompted for details and clarification where necessary. This included descriptions of any technical support they received, the process of logging in, and activities they engaged in during the session. Other questions focused on perceived benefits and drawbacks of in-home video visits, prior use of technology, and caregiver preference for care modality (e.g., in-clinic or video).

Caregivers were also asked to quantitively rate their level of involvement with the patient’s care using an item from the *Caregiver Health Engagement Scale*, specifically, “I actively collaborate with my loved one’s health professionals” [47]. They also rated their technological literacy using an item from the *Assessing Computer Anxiety* scale, specifically, “I am confident in my ability to use computers” [48]. Both items were rated on five-point Likert scales of agreement with higher ratings indicating stronger agreement.

Interviews were audio-recorded and transcribed using Microsoft Teams. Transcripts were reviewed and cross-checked with audio-recordings and amended for accuracy before analysis.

### Data Analysis

This study employed a directed content analysis [49] utilizing a rapid analytic approach [50,51], given our project’s 1-year timeline. Analysis focused on identifying specific details around caregivers’ involvement in video telehealth. Rapid analysis within a time constrained project offers significant advantages by facilitating timely, actionable decision making while preserving analytical rigor [50]. Specifically, we conducted qualitative analysis using RREAL sheets, which are working documents developed by the Rapid Research and Evaluation Lab (RREAL) that allow for data synthesis while data collection is ongoing [51]. RREAL sheets are structured, thematic templates for real-time data transfer. The RREAL sheet (which we organized using MS Word^®^) was utilized to organize themes related to our study objective, understanding the experience of caregivers who participated in an in-home occupational therapy video visit. To ensure consistent synthesis, team members (MG and DW) used RREAL working documents [35], structured templates that support real time data summarization. We created MS Word^®^ templates aligned with analytic domains and interview questions to capture key findings, quotes, and observations.

Each transcript was read and analyzed by two study team members (MG and DW), during which each independently entered data into the RREAL sheet which was organized by interview guide question. MG and DW then met to discuss completed RREAL sheets related to each interview and to reach consensus on thematic areas. We established clear rules for populating these templates, beginning analysis before all interviews were complete. In regular team meetings, we then consolidated individual sheets into a consensus version, maintaining version control and synthesizing data across participants. Using constant comparison [49], we reviewed patterns with analysis concluding when we reached consensus on our final list of themes.

## RESULTS

Below please find participating caregiver demographic details and characteristics, as well as those for the patients for whom the video visit was conducted. We also present descriptions and supporting details for our five themes: (1) Caregiver Level of Involvement in In-Home Video Telehealth Visits; (2) Experience of Technological Aspects of In-Home Video; (3) Experience of Clinical Aspects of In-Home Video; (4) Beliefs about the Usefulness and Scope of In-Home Video; (5) Recommendations for In-Home Video.

### Caregiver and Patient Characteristics

See Table 1 for participant characteristics. Caregivers were all White, mostly female (94.7%), with an average age of 60 (range 32 to 77). Regarding relationships of caregivers to patients, caregivers were commonly the patient’s spouse (42.1%) or child (42.1%). Patients were White, mostly male (94.7%), with an average age of 78 (range 66 to 88). Most caregivers (68.4%) lived with the patients. Nearly all caregivers described themselves as actively involved in the patient’s care, with 94.7% agreeing or strongly agreeing with the statement that they actively collaborate with their loved one’s health professionals. Caregiver confidence with technology varied, but most (68.4%) agreed or strongly agreed with the statement that they were confident in their ability to use computers, with only three (15.8%) disagreeing. According to visit notes within patients’ electronic health records, the occupational therapy in-home video visits which caregivers assisted with focused on provision of Durable Medical Equipment (DME), including tub transfer benches, raised toilet seats, and stair lifts (68.4%), or home safety assessments (21.1%), with the remaining (10.5%) being general follow-up visits.

### Caregiver Level of Involvement in In-Home Video

According to interviews, caregivers’ level of participation in the video visits varied, with some caregivers encouraging patient engagement by stepping back during the visit and others taking a more active role. Some caregivers intentionally tried to let the patient take the lead, as with one caregiver who stated, “I try to be quiet.” At times patients only needed help with technology, such as providing for the requirements of video like a video-enabled device or email. One caregiver stated, “if it’s something like where it has to go through an e-mail… that’s where she has an issue because she doesn’t have an e-mail.” Others assisted because of the patient’s lack of technology and technical skills, as when a caregiver noted, “I had to because my dad only has a flip phone and he’s 88 years old and he does not know anything about computers.” Sometimes caregivers assisted despite their own technological challenges: “I’m pretty limited with my expertise with electronics, but he’s even more limited.” Caregiver involvement was often essential for patients with cognitive or other challenges such as hearing loss or low frustration tolerance, as when a caregiver noted, “ he doesn’t have the patience. I’ve got more patience with it.” Caregivers frequently acted as intermediaries, helping patients communicate effectively and ensuring accurate information is shared with the care team. One caregiver noted, “I’ll have to repeat what they’re saying, ‘cause he’s hard of hearing.” Another remarked (of her husband with memory complaints), he “kind of fills in… What he thinks might have been, which is not a good situation.” Having patients hold the device during video telehealth appeared to engage more passive patients.

### Experience of Technological Aspects of In-Home Video

Caregivers’ description of the technological aspects of in-home video revealed the centrality of their role, particularly in the presence of technological challenges. Caregivers assisted with several technical tasks—logging in, activating microphones, or issues with internet connectivity—which were common but often perceived as minor. One caregiver stated, “Sometimes we get disconnected several times… and then we get connected back.” Challenges at times related to the initial log-in (which involved using VHA’s proprietary videoconferencing application). One caregiver noted, “Getting the app and putting it on the phone. That was the hardest part.” Some caregivers who discussed having received technical test calls prior to video felt they helped to feel prepared to troubleshoot potential issues. Others had a test call but still struggled with technology, with one caregiver noting, “I didn’t know how to get my camera and microphone turned on on the computer because somehow or other it had gotten turned off and I didn’t know how to get it back on again.” Some caregivers managed well with written instructions alone.

### Experience of Clinical Aspects of In-Home Video

Caregivers often assisted with clinical aspects of in-home video visits, particularly for complex assessment processes. Caregivers assisted with DME or home safety assessments through camera positioning to enable visualization of the home or the patient. One caregiver described using reverse camera during an assessment for a stair lift for his mother in which the clinician “asked us to show her what our stairs look like because it was all for getting a new chair lift put in… so I just flipped the screen on my phone around and showed her [the OT] the steps and then my mom walked up the steps like she normally does in the video.” This caregiver also took measurements of the width, height, and length of the stairs. Another caregiver described an assessment for equipment to assist with her spouse’s mobility problems, stating, “I showed her like how small the apartment was, not the complete thing but the living room and the bathroom.” Others helped set up the environment so it was more conducive for a medical visit, with one caregiver stating, “we turn the TV off... just get it to where it’s an acceptable level of background noise.” Such caregiver assistance was especially important for patients with mobility challenges which limited their capacity to assist with the assessment, as when one caregiver noted, “he walks with a walker, so it would be hard for him to walk around and hold the camera at the same time.” Caregivers also assisted when the patient was the focus of the assessment and could not video him or herself.

### Beliefs about the Usefulness and Scope of In-Home Video

Caregivers had mixed perceptions about the usefulness and scope of video telehealth for certain types of services. The usefulness of telehealth often related to convenience for caregivers and patients, particularly older patients with complex care needs. Several caregivers described the benefit of not having to drive long distances to receive care, as when a caregiver described, “if we got an appointment in Gainesville at 9:00 o’clock, that means we have to leave here about four or five hours before that to give us plenty of time to get there... Then we have to drive back, and then sometimes we don’t get home till 6–7 o’clock at night.” Travel was at times burdensome because of patients’ medical complexity, as when a caregiver stated, “when she goes down to the VA, it’s an all day scenario for her. And by the time she gets home, she’s wiped out.” Another discussed the dangers of travel given her husband’s continence issues, noting, “he’s been having trouble with his kidneys, his bladder. And sometimes we’ll have to stop… And the last time we went, he didn’t make it hardly in time. And you know it’s kind of a little embarrassing for him.” Similarly, a spousal caregiver related, “if anything happened like to the car or something and we were stuck on the side of the road, my husband’s health is not that good that we can sit there in the cold, you know, and wait for somebody to come and help us.” An adult child caregiver discussed the ease of video compared to bringing both her patient father and her mother to in-clinic appointments, stating, “It’s been made a little easier on me because like I said, taking both of them out by myself is pretty much impossible right now.”

Caregivers’ general attitudes toward in-home video telehealth also appeared to relate to their own use and comfort with technology. Some caregivers who used technology regularly for other purposes disliked it for medical care, finding it impersonal, burdensome, or cost-cutting. One caregiver remarked, “I’m doing all this stuff to provide you with all the information and where’s my part of the cut?” Others—especially those less familiar with technology—felt anxious but had been willing to try it, even for their own medical care. One caregiver who had not used video for her own medical appointments noted, “No, I usually only go once a year and there’s no reason to, I mean, it’s just a yearly physical so I don’t, they don’t usually do video visits for that…but yeah, I would give it a shot, I’m not against it.” Factors like access to care influenced perceptions of video telehealth but not always in the direction one would expect. For example, one caregiver of a patient with complex medical challenges stated that she would rather “have him go to the appointments if the weather’s decent and everything and he’s feeling OK cause I don’t wanna make any more homebound than he is.”

Related to the scope of telehealth services, caregivers perceived in-home video as appropriate with some limitations, particularly for diagnostics and services requiring hands-on care. Caregivers believed that in-clinic appointments focused on discussion could easily translate to video, with one caregiver noting, “sometimes we’ve gone up there and all they’ve done is talk to them and I thought, Gee, I wish we could have done this by video.” Some caregivers believed that video visits cannot capture the same level of detail as in-person appointments, particularly for conditions that require close visual inspection, such as skin issues, or when the video view is limited. One caregiver stated, “you can’t always see what is wrong unless you see it up close.” Similarly, acute issues were perceived as not suitable for in-home video telehealth, with a caregiver noting, “If it’s not a dire situation, I think it can be handled over telehealth.” In-home video telehealth was generally viewed as inadequate for physical exams, lab work, and certain procedures. One caregiver stated, “Sometimes you just have to go in. He had some nerve test done. We couldn’t do that over the phone.” In-person visits were also preferred because they allow for hands-on interventions, as when a caregiver stated, “The only drawback [with video] is that the doctor wouldn’t be able to physically touch him.”

### Recommendations for In-Home Video

Caregiver suggestions for how to optimize in-home video telehealth related to improving resources and assistance for patients and caregivers. Caregiver recommendations included the suggestion that an employee or volunteer could assist patients who lacked a caregiver or when patients’ caregivers cannot manage technology. Caregivers also suggested that accommodations like headphones, transcription, or tablet stands may enhance the experience. One caregiver recommended that paid in-home staff, such as home health aids, could assist with video, noting, “if she was here during the week and I wasn’t able to navigate it, the videos, if she was able to, she is part of his health care team. Could she be utilized?” Recording the in-home video telehealth session was also suggested, “for cognitive reasons or whatever. If the video appointment could be recorded and then we could watch it back and he could ask questions or he could add something that he didn’t think about during the appointment.” Another caregiver suggested simplifying the VHA’s video telehealth platform to be as user-friendly as FaceTime, noting, “if an elderly [person] can at least answer their iPhone on a FaceTime, that would definitely be way easier, of course, because you just literally push the button and answer the phone.” Caregivers often offered suggestions in the absence of experiencing challenges themselves, acknowledging that in-home video telehealth may poses difficulties for others.

## DISCUSSION

To our knowledge, this study is the first to gather caregivers’ perspectives of assisting an older rural patient with an in in-home video telehealth occupational therapy visit. Understanding the caregiver experience supporting patients to access occupational therapy via in-home video is vital to optimizing this service delivery option for patients who need it the most. It also has potential significance for clinical care more broadly. Below we summarize key findings and relevant take-aways to guide future work.

Caregivers supporting older rural patients to engage with in-home video telehealth were mostly female and patients’ spouse or adult child, which aligns with national data about Veteran patients’ caregivers being mostly female spouses (since Veterans are mostly male) [52]. The high proportion of adult child caregivers in our study aligns with evidence supporting their increased role supporting rural Veterans compared to those in urban settings [52]. Our finding that caregivers are actively involved in patients’ health care aligns with evidence describing their central role in assisting with the day-to-day care needs of Veterans [53]. This includes our own work examining the caregiver support role in occupational therapy in which clinicians observed that caregivers were particularly helpful assisting older patients, especially with technological aspects [31,54,55]. Relatedly, the high involvement in patients’ care that caregivers of this study reported also aligns with evidence emphasizing the need for care teams to integrate caregivers into patients’ care [56].

According to our interviews, caregivers are a technological bridge between clinicians and patients by supplying devices, helping with log-in, and managing the technical aspects of video visits. Caregiver involvement is often critical for patients facing cognitive, hearing, communication, or technical skills challenges that prevent them from independently navigating video visits. This aligns with evidence from our work specific to caregivers’ technical support role in occupational therapy video telehealth [32,45] and occupational therapists’ perspectives on the importance of caregivers to support older patients in accessing telehealth [57]. Our finding that many caregivers take care to ensure the patient remains engaged during sessions through thoughtful camera placement aligns with evidence revealing the importance of caregivers to facilitate patient participation in video telehealth [58]. Regarding caregivers’ clinical support tasks, our finding that much of the caregiver support role during in-home occupational therapy video related to home safety and Durable Medical Equipment evaluations aligns with our prior work from occupational therapists’ perspectives [31,45]. More research is needed to explicate the specific tasks caregivers engage in during in-home video telehealth for occupational therapy and during assessments more broadly.

Our findings also highlight that for rural or homebound Veterans, in-home video may be the only feasible way to receive specialty care, which speaks to access barriers for occupational therapy [45] and other services like palliative care [59]. The fact that some caregivers prefer in-person visits as an opportunity for patients to leave the house aligns with extant data revealing that preferences are nuanced and may vary by context and service; patients may choose telehealth to avoid infection or for cost-savings but prefer going into the clinic for diagnostics and physical examinations [60]. Related to caregivers’ perceptions of the use of in-home video for specific services, extant data from caregivers of homebound older adults perceive equivalence between in-person and video for mental health [58]. Our finding that caregivers perceive in-home video as limited for more complex evaluations, diagnostics, or acute situations aligns with evidence endorsing the superiority of traditional care for accurate diagnosis and management [60], physical examination [61], and procedures involving visualization [62]. Related to occupational therapy services in particular, perceived limitations for in-home video are echoed by practitioners themselves [63], which speaks to a gap in adapting more complex assessment processes to in-home video.

According to caregivers, in-home video telehealth offers significant advantages for older rural Veteran patients by reducing travel time and logistical challenges, particularly for patients who live far from VHA facilities, lack reliable transportation, or face severe weather conditions. This aligns with several studies highlighting the benefits of in-home video generally [62,64], and for rural family caregivers in particular [65]. In-home video can also ease the burden on caregivers who may be balancing work, childcare, or their own health needs [65]. For patients with mobility limitations, chronic pain, or cognitive impairments that make in-person visits stressful, caregivers also reported that in-home video is a more comfortable and accessible alternative. This aligns with our prior work demonstrating benefits of video on preserving patients’ daily routines in the context of dementia [66]. Our finding that in-home video enhances caregiver engagement in patient care by offering greater scheduling flexibility aligns with evidence endorsing key benefits of in-home video to overall convenience and family-centered care [67,68].

Related to our finding about varying caregiver confidence with technology, while there is limited data specific to caregivers and confidence with in-home video telehealth, a recent study found that many caregivers of patients with dementia lack confidence with digital health tools despite using them regularly [69]. Caregivers of adults with chronic illness and adults with disabilities also report digital literacy gaps and training needs for digital tools [70,71]. Similarly, our finding that caregivers may be only modestly more technologically skilled than the patients they support and may require help navigating the video platform aligns with extant data [72]. This highlights the need to integrate caregivers in development and implementation of telehealth tools as well as training and support resources [73]. Care teams and clinicians can also formally assess patient and caregiver experience with video-based technology, such as through a brief measure recently developed by VHA researchers [74]. Caregiver recommendations to simplify video telehealth technology for those comfortable with familiar platforms like FaceTime aligns with evidence about an increased learning curve for novel software [75]. Our finding that some caregivers face connectivity challenges reflects broader infrastructure challenges including a lack of reliable broadband in rural areas compared to urban settings [52].

Also specific to older patients, there may be a relationship between caregiver burden and openness to telehealth [76]. This underscores the importance of assessing not just caregivers’ technological readiness but also their overall burden, perhaps through administration of a standardized measure such as the Zarit Burden Interview [77]. It is also important to note that spouses and adult child caregivers have differing levels of burden [78] which may contribute to their ability and willingness to engage in in-home video telehealth.

## CONCLUSIONS

In sum, in-home video telehealth plays a crucial role in expanding access to occupational therapy for older rural patients who face significant obstacles to in-person care, including distance, mobility limitations, and health-related challenges. Caregivers are central to making these visits possible, providing both technical and practical support that enables patients to participate effectively. Though caregivers may be willing to participate in video, their perspectives about video are nuanced. While caregivers acknowledge that video visits work well for routine follow-ups and check-ins, they also recognize clear limits for more complex or urgent needs, which speaks to the need to develop standardized procedures for these services adapted for in-home video. Despite the telehealth infrastructure of the Veterans Health Administration, barriers such as insufficient connectivity for video persist, speaking to large-scale resource needs. Even so, caregivers often view in-home video telehealth as a convenient, valuable option that supports continuity of care and reduces strain on everyone involved.

## IMPLICATIONS FOR PRACTICE

Caregivers play a critical role in enabling older rural patients to participate in in home occupational therapy video telehealth. The following practice considerations may help to optimize care delivery:

- Integrate caregivers intentionally by clarifying their role before visits and involving them as partners in communication and care planning.
- Provide simple, accessible technical preparation, including optional test calls, clear written instructions, and (where possible) user-friendly platforms to reduce technological burden.
- Offer structured guidance for clinical tasks such as camera positioning, environmental setup, and home measurements to improve accuracy during home safety and DME assessments.
- Consider using video strategically for routine or non-urgent visits while helping families understand when in person care may be needed for hand-on evaluation or complex concerns.
- Assess caregiver readiness and burden, including technological comfort, to tailor support and avoid over-reliance on caregivers with limited capacity.
- Try to address rural access barriers, including connectivity limitations and device needs, to ensure equitable telehealth delivery.

## LIMITATIONS

This study has several limitations. First, our findings may not generalize beyond the Veterans Health Administration, given the VHA’s unique telehealth infrastructure and specific Veteran factors. Veteran and caregivers were also racially homogenous, which may limit applicability to non-White populations. In addition, this convenience sample included caregivers who had participated in a successful video telehealth visit, and therefore findings may not apply to either non-users of video or to those who attempted video but were unsuccessful. Future work should include a broader range of perspectives in larger scale studies, including patient perspectives where possible.

## Figures and Tables

**Table 1.** Participant Characteristics.

ID	CG Age	CG Lives with Patient	CG Involvement in Patient Care^1^	CG Confidence with Computers^2^	Patient Age	Patient Rurality	Caregiver Relationship to Patient

1	57	No	Strongly Agree	Strongly Agree	88	98%	Child
2	38	No	Strongly Agree	Strongly Agree	79	99%	Child
3	72	Yes	Strongly Agree	Strongly Agree	79	97%	Spouse
4	65	No	Strongly Agree	Agree	83	96%	Child
5	61	No	Agree	Agree	88	96%	Child
6	61	Yes	Strongly Agree	Neutral	72	100%	Spouse
7	53	No	Strongly Agree	Strongly Agree	76	95%	Child
8	64	Yes	Strongly Agree	Strongly Agree	73	96%	Spouse
9	32	Yes	Neutral	Strongly Agree	71	99%	Child
10	72	Yes	Agree	Neutral	79	97%	Spouse
11	69	Yes	Strongly Agree	Disagree	87	98%	Spouse
12	56	Yes	Strongly Agree	Agree	68	96%	Sister-in-law
13	66	Yes	Strongly Agree	Disagree	66	98%	Spouse
14	51	Yes	Strongly Agree	Neutral	87	98%	Niece
15	71	Yes	Strongly Agree	Disagree	74	97%	Spouse
16	58	No	Strongly Agree	Strongly Agree	84	98%	Child
17	53	Yes	Strongly Agree	Strongly Agree	77	97%	Niece
18	77	Yes	Strongly Agree	Strongly Agree	65	96%	Spouse
19	66	Yes	Strongly Agree	Strongly Agree	87	96%	Child

1 Response to statement, “I actively collaborate with my loved one’s health professionals,” from *Caregiving Health Engagement Scale* (CHE-s) [47], on five-point Likert scale of agreement.

2 Response to statement, “I am confident in my ability to use computers,” from the *Assessing Computer Anxiety* scale [48], on five-point Likert scale of agreement. CG = Caregiver.

## Data Availability

A deidentified dataset of the study is available from the authors upon reasonable request.

## APPENDIX A. CAREGIVER INTERVIEW GUIDE.

### Caregiver Interview Guide

#### After recording has begun.

Thank you for agreeing to participate. As I said, I am recording this interview so I can go back and review our conversation later. The recording will be kept private. Do you agree to participate and to have this interview recorded? [**No** >> Thank you.] [**Yes** >> Great.]

To reiterate the purpose of these interviews, we are interested in learning about use of VA Video Connect, which is VA’s version of FaceTime or Zoom, to deliver OT services into the home. We hope to gain the experiences of people like you who assist Veterans with video calls.

This is for a project funded by the Office of Rural Health, the goals of which are to understand how to improve video OT services to best meet the needs of Veterans and their caregivers. We want to hear ‘the good, the bad, the ugly’—what worked well and what did not.

Before we get started, do you have any questions?

First off, we have some general questions about you and your relationship to the Veteran:

**Table:**

Question	Answer
*Caregiver Demographics*	
1. Caregiver Age (at time of interview)	• _____years • prefer not to answer
2. Caregiver Gender: (Circle one)	• Male • Female • Prefer to self-describe________________ • Prefer not to answer
3. Caregiver Race: (Circle all that apply)	• White • Black or African American • Native American or American Indian • Asian/Pacific Islander • Other___________________ • Prefer not to answer
4. Caregiver Ethnicity:	• Hispanic or Latino • Not Hispanic or Latino • Prefer not to answer
*Caregiver and Veteran Details*	
5. What is your relationship to the Veteran?	Veteran is my: • Spouse • Parent • Sibling • Family Member • Friend • Acquaintance • Employer • Other_________________
6. Do you live with the Veteran?	o Yes o No o ***If no,*** ▪ Who lives with the Veteran? ▪ About how far from the Veteran do you live? Miles_______/Minutes_______(RURAL)
**Now I wanted to ask about your involvement with the Veteran’s care**	
7. I actively collaborate with my loved one’s health professionals *(Caregiving Health Engagement Scale)*	5-strongly agree 4-agree 3-neutral 2-disagree 1-strongly disagree
**Now I had a question about your confidence using technology. Please rate on a scale of 1 to 5, with 1 being “strongly disagree” and 5 being “strongly agree”**	
8. I am confident in my ability to use computers. *(Assessing Computer Anxiety scale)*	5-strongly agree 4-agree 3-netral 2-disagree 1-strongly disagree

Now I want to ask you some questions about YOUR use of technology and experience with video.

- Tell me about YOUR use of technology. How often do you use computers or tablets? Do you go online or use the internet?
- Have you ever had a video visit such as Facetime or Zoom with a friend or family member for work or personal reasons?
  - Yes
    - What were some of the reasons you decided to use video?
    - What platform did you use? (Zoom/Facetime/Google Duo)
    - Tell me about that experience. Did you need assistance getting it set up? What did you like about it? What did you not like about it?
  - No
    - If applicable, why or why not?
- Have you ever had a video visit with a medical provider?
  - Yes
    - What platform did you use? (Zoom/Facetime/Google Duo)
    - What were some of the reasons you decided to use video?
    - Tell me about that experience. What did you like about it? If yes, what did you think of that experience?
  - No

Now I would like to ask about your experience of the video/VVC OT telehealth session for your Veteran. If necessary, remind caregiver of visit details.

- What were your expectations for the video/VVC session? (Prompt: Visit purpose, how long it would take, their role)
- What if any preparation did you have to make to attend the video visit?
- What preparation or training did you receive from VHA or the OT prior to the session? If none, would training or assistance have been helpful?
- Tell me about your experience of the visit.
  - Present the whole time or not?
  - Location: Veteran’s home, other
  - What type of device did you use? Veteran’s technology or caregiver’s?
  - Technology—did they assist with tech set-up? How did that go?
  - Tech challenges with set-up or during visit? How was the service?
  - What was your experience of the OT’s clinical care? Did you have to provide any hands-on assistance during the visit?
- How did the OT VVC session compare to coming into the hospital?
- Was there anything that made it difficult to participate in the OT VVC session?
- Was there anything you liked about it?
- What would have helped?
- What would you recommend to improve video/VVC?
- Is there anything else you would like to add?

Thank you so much for your time.

## ABBREVIATIONS

VHA: Veterans Health Administration
OT: occupational therapy
OTKI: Occupational Therapist Key Informant
